# Just do it! Study time increases mathematical achievement scores for grade 4-10 students in a large longitudinal cross-country study

**DOI:** 10.1007/s10212-021-00546-0

**Published:** 2021-04-07

**Authors:** Markus Wolfgang Hermann Spitzer

**Affiliations:** Institute of Psychology, Albert-Ludwigs-Universität Freiburg, 79085 Freiburg, Germany

**Keywords:** Study time, Mathematics, Academic achievement, E-learning

## Abstract

Decades of research produced inconsistent findings on whether study time can lead to achievement gains in mathematics. Data generated by more than six thousand students from three different countries who solved more than 1.1 million problem sets using a dedicated mathematics software are analyzed regarding the effect of study time on students’ achievements in mathematics. Results showed that more study time led to higher performance scores in mathematics. Further analyses revealed that low-performing students in the first school year (2017-2018) who increased their study time in the following year (2018-2019) revealed greatest gains in performance in the same school year (2018-2019) and even in the year after (2019-2020). Finally, results replicated previous observations of robust performance scores within students over the three school years, with performance scores in 2017-2018 predicting those of 2018-2019 which predicted those of 2019-2020. These results support the idea that students, in particular low-performing students, can boost their academic abilities to upper levels when increasing their study time.

## Introduction

Children and adolescents who reach high levels of mathematical abilities early in life persist on these levels over time (Aunio and Niemivirta, 2010; Jordan et al., 2011; Siegler et al., 2012; Watts et al., 2014). Furthermore, strong mathematical abilities at young age are related to a higher socio-economic status and higher salaries during adulthood (Murnane et al., 1995; Ritchie and Bates, 2013; Rivera-batiz, 1992). However, the number of students who do not achieve ordinary mathematical skills at the end of high-school is troubling (Moore et al., 2010). These observations raise the challenge on the best way to boost mathematical abilities in poorly performing children and adolescents (National Mathematics Advisory Panel, 2008).

One major unresolved question in the search for factors that promote students’ mathematical abilities is whether increases in study time lead to gains in mathematical achievement scores. While decades of research have demonstrated that the accumulated hours spent with deliberate practice determined successful acquisition of knowledge and skills in other domains, such as for professional violinists (Ericsson et al., 1993), chess players (Krampe and Ericsson, 1996), and soccer players (Roca et al., 2012; Ward et al., 2007), results from studies on whether study time leads to increased knowledge and achievement scores in education are inconsistent (Jez and Wassmer, 2015; Macnamara et al., 2014; Pittman et al., 1986; Plant et al., 2005; Schuman et al., 1985). This provided the motivational background for the present investigation of the effects of study time on mathematical achievement scores using a large longitudinal dataset from three different countries. In the following sections, we overview literature on effects of study time on academic achievement scores.

### Effects of study time on academic achievement scores

Plant et al. (2005) observed that prior abilities and self-regulation skills, but not study time, influenced grade point average scores of college students. Furthermore, they observed that the quality of study time differed between students (Plant et al., 2005; for similar results see Schuman et al., 1985). Another study reported that effort and not study time predicted academic outcomes best (Flunger et al., 2015). A recent meta-analysis revealed that study time only explained 4% of the variance of educational outcomes (Macnamara et al., 2014). Moreover, the importance of self-regulatory skills on achievement scores have been highlighted by others (Schunk and Zimmermann, 1997; Zimmerman and Kitsantas, 2005). In line with these observations, no significant difference between students’ academic achievement scores was observed for students with a reduced academic school year of 20 days, compared to students with a regular school year (Pittman et al., 1986). In contrast to these findings, Jez and Wassmer (2015) reported that study time had a positive impact on high-school students’ academic achievement scores, and in particular, that low-performing students benefit the most from extra study time (Jez and Wassmer, 2015). On a similar account, Doumen et al. (2014) report that study time influenced course grades of college students (Doumen et al., 2014). In addition, a positive correlation between grand average college scores and study time (Gortner-Lahmers and Zulauf, 2000) and the positive effects of deliberate practice on academic achievement scores have been reported (Eskreis-Winkler et al., 2016).

Whereas the dependent variable in these studies was grand point average scores, others narrowed their research on the effects of study time specifically on mathematical achievement scores of middle school students. For example, several studies reported a positive correlation between study time on homework assignments in mathematics and learning achievements (Cheema and Sheridan, 2015; Cooper et al., 2006; Cooper and Valentine, 2001). However, others did not observe a positive effect of homework study time on students’ achievement gains in mathematics (Flunger et al., 2015; Trautwein, 2007). Several other studies argue that the quality of study time and not just the quantity influence the effect of study time on mathematical achievement (Perels et al., 2009; Rosário et al., 2013). For instance, Rosário et al., (2013) investigated the effects of study time on mathematical achievement in 1300 middle school students. They observed that factors such as self-regulatory learning scores and motivational factors mediated the effect of study time on mathematical achievement.

In sum, the literature yields inconsistent results regarding the effect of study time on mathematical achievement gains. While few studies observed a positive correlation between study time and achievement scores, others observed that the positive effect of study time is mediated by factors such as self-regulatory skills and motivational factors (Rosário et al., 2013), effort (Flunger et al., 2015; Schuman et al., 1985; Trautwein, 2007), and the quality of study time (Perels et al., 2009; Rosário et al., 2013). Furthermore, it is not yet clear whether more study time alone can enhance the achievement scores of low-performing students, and whether these gains of low-performing students’ due to increased study time are sustainable and persist over longer periods of time.

From a methods point of view, it appears important to note that the studies reviewed above investigated the influence of study time, by means of a questionnaire, on academic achievement scores on tests after the study time. Given the generally poor reliability of self-reported questionnaire estimates, it may be better to assess study time directly with the use of computer-based measurements of study time. In the following section, we outline advantages of online learning environment when measuring study time and achievement scores.

### Online learning environments enrich measurement possibilities

A major advantage of online learning environments is the possibility to analyze massive amounts of collected data with precise measures such as the time students spent completing problem sets, students’ results on these problem sets, and the date on which problem sets were completed (Koedinger et al., 2007; Koedinger et al., 2015). In addition, these data can be analyzed within long-term investigations on how students’ mathematical abilities develop over years of schooling, and which factors influence these learning trajectories (Baker and Inventado, 2016).

For instance, Louw et al. (2008) made use of these technological advantages and analyzed the data collected with a dedicated mathematics software package. They examined the achievements in mathematics of students from three different schools using a mathematical software over several months. Thereby, they were able to show a positive correlation between amount of study time with the software and students’ improvement scores between grades eleven and twelve (Louw et al., 2008). However, the authors noted that software usage time was generally very low, with an average of less than 3 h of math training spent with this software within 5 months. Moreover, their results do not distinguish between students with weak mathematical abilities and students with strong mathematical abilities. Therefore, it remains unclear if such a training approach can be specifically beneficial to weaker students. In this investigation, we made use of a software learning tool for mathematics which shall be briefly described.

### The Bettermarks software

We analyzed the data collected with the software package Bettermarks within the time frame of three consecutive school years (year 1: 2017/2018; year 2: 2018/2019; year 3: 2019/2020). The Bettermarks software is a mathematical learning software that is currently (as of March 2020) used as a complement or even supplement to traditional math books in over 600 schools and over 5000 classes across Germany, Uruguay, and the Netherlands. The software comprises over 100 “book topics” (i.e., general topics, such as “Basics calculations of fractions,” or “Advanced calculations of fractions”) that comprise over 2000 different problem sets from classes 4-10 in four different languages (German, English, Spanish, and Dutch), with the highest usage in Germany, Uruguay, and the Netherlands. Each of the book topics was created in close relation to the curricular of each country, respectively. Put in other words, book topics represent the curricular of the school system in each country, respectively. Each book topic consists of an introduction page to the mathematical topic and 12 problem sets on average and each of these problem sets consist of eight distinct mathematical problems on average. Bettermarks is used within the class context, meaning that teachers assign problem sets to students within the software (just as they would with traditional math books). Students compute these assigned problem sets and receive immediate feedback on their result (correct/incorrect) for each problem. Furthermore, they receive the average score they reached on the particular problem sets after computing all individual problems in a problem set. For example, if a student computes 10 single mathematical problems within a problem set and gets 8 out of 10 answers correct, he receives feedback that his/her average score was 80%. Students are able to repeat a problem set; however, the parameterization changes on the next attempt. This new parameterization is necessary, because students could memorize the result of the first attempt to cheat on the new attempt by inserting the memorized answer of the system. Teachers are able to monitor the results of their students and are able to incentivize their students to perform as accurately as possible on the system by including the results in students’ oral grades. Students do not see the problem set before they select to compute them and thus, the process of selecting a problem set allows the precise measurement of the timepoint when students start to work on a mathematical problem set. When students finish the calculation of all single problems within a problem set, they press a proceed button, allowing the precise measure of the end timepoint of computing the problem set. Together, the software allows the collection of data on study time of problem sets and students’ performance on these problem sets in relation to other students’ performance on the same problem sets (see the “Data analysis” section for a detailed description on the performance measure).

### Purposes of this study

Taken together, the reported studies suggest the need for a systematic investigation on study time on mathematical achievement in order to answer the following questions:

- Does study time in itself have a positive effect on mathematical achievement scores in K-12 students?
- Does study time boost performance for low-performing students in particular?
- Do increases in performance, due to more study time, persist over time?
- Does the quality of study time matter?

To find answers for these questions, we analyzed the average study time and the average performance score on problem sets students computed within three consecutive school years in three distinct analyses (see Table 1). We first analyzed whether students’ study time in year 1 influenced their performance scores in this school year (analysis 1). Another analysis (analysis 2) was carried out to replicate the effect of study time on performance in year 2. In addition, this analysis investigated whether students’ performance in year 2 depended on the performance of the previous school year (year 1) and whether this dependency would interact with the amount of study time during this school year (year 2). In a final analysis (analysis 3), we tested whether students with a low performance in year 1, who increased their study time in year 2, also increased their performance in year 3. Furthermore, we examine whether the effect of study time differed between students who did improve, compared to students who did not improve while studying. More precisely, we tested whether low-performing students in year 1, who increased their study time in year 2, and did improve in year 2, varied in terms of their performance in year 3, from those low-performing students in year 1, who increased their study time in year 2, but did not improve their performance in year 2.

**Table 1:** Dependent and independent variable(s) of each conducted analysis

Analysis ID	Dependent variable	Independent variable(s)
Analysis 1	Performance_Year 1_	Time_Year 1_
Analysis 2	Performance_Year 2_	Time_Year 2_, Performance_Year 1_
Analysis 3	Performance_Year 3_	Time_Year 2_, Performance_Year 1_, Performance_Year 2_

*Note:* The main effects and all possible interactions were included in the linear regression models

We tested each analysis with students who used the software in Germany. Each analysis was also carried out for two further datasets collected from Uruguayan students, and Dutch students. The purpose of these replications was to test the robustness of the results obtained from the data from German students.

## Method

### Software

The Bettermarks software has been used in classrooms since 2008. The learning software allows teachers to assign problem sets from different book topics to students. Teachers use the software like a textbook inside the classroom and for homework assignments and they can freely choose which problem sets they want assign to the students of their class. Over 100 book topics cover over 2000 different problem sets of the curricula for students from grades 4-10 (age range: 9-16). The software can be used as a complement or supplement to traditional math books. High-school teachers use the software to either assign problem sets to students as homework, or as exercises within the classroom. The data from classroom or homework assignments were analyzed in this research project. The data is stored anonymized and thus any information relating the students with the data (e.g., students’ gender or age) cannot be obtained.

### Inclusion criteria

The research project included data of students from three different countries (Germany, Uruguay, and the Netherlands) who used the software from September 1st, 2017, until March 1st, 2020, respectively. In addition to this time frame, five further inclusion criteria were applied. Data analysis only included (a) students from classes with more than 20 students; (b) students who used the software in each school year; (c) students who computed at least 50 distinct problem sets with the software; (d) answers from completed problem sets^1^; and (e) problem sets completed by at least 30 students. These inclusion criteria were set prior to the data analysis and only the data of students who met these inclusion criteria was obtained from Bettermarks.

The obtained dataset comprised 4090 German students who computed a total of 406,396 problem sets, 351 Uruguayan students who computed 38,970 problem sets, and 1690 students from the Netherlands who computed 713,929 problem sets. In sum, the current research project comprised a total of 6131 students who computed a total of 1,159,295 problem sets.

### Independent and dependent variables

Three independent variables described the study time (in hours) spent computing mathematical problem sets with the software for each student in each school year (measured from September 1st, until August 31st) labeled as time_Year 1_, time_Year 2_, and time_Year 3_ (interval scaled)^2^. In addition to these study time variables, we computed students’ performance on each problem set as dependent variables. The performance was calculated as follows. On each problem set, students achieved an accuracy score between 0 and 1. However, this accuracy score depended on the difficulty of the problem set. To account for problem set difficulty, we computed the average accuracy score of each problem set from results of all students (which we refer to as the population) on each problem set. For instance, if 30 students computed a particular problem set with an average accuracy of 80%, this accuracy score was then used as an estimate for problem set difficulty. The performance of each student was then calculated as students’ accuracy on a problem set minus the difficulty of this problem set. Positive performance scores indicate a result above the average accuracy of the population while negative performance scores indicate a result below the average accuracy of the population. Finally, the average performance scores of each student in each time window were computed and labeled as performance_Year 1_, performance_Year 2_, and performance_Year 3_ (interval scaled).

### Data analysis

Effects were estimated with a linear regression model in the R environment for statistical computing (R Core Team, 2013; RStudio Team, 2015). Graphs were plotted with the *sjPlot* package (Lüdecke, 2020).

A linear regression estimated the influence of the independent variable time_Year 1_ on the dependent variable performance_Year1_ on German students, Uruguayan students, and Dutch students (analysis 1). We expected that students with increased study time revealed increased performance scores in all three analyses.

In the following analysis (analysis 2), we investigated the stability of students’ performance in mathematics between school years and whether the amount of study time in the following school year influenced students’ performance in this year. Therefore, we utilized students’ performance score of the first year (performance_Year 1_) as an independent variable and examined its effect on the performance score in the following year (performance_Year 2_). In addition, we included the independent variable time_Year 2_ to replicate the result of analysis 1. Finally, the interaction between the independent variables performance_Year 1_ and time_Year 2_ was added in the linear regression model. Again, the analysis was conducted for German students, Uruguayan students, and Dutch students. We expected that the performance_Year 1_ had a positive effect on the performance_Year 2_, with students with low-performance scores in year 1 having low-performance scores in year 2 and students with high-performance scores in year 1 having high-performance scores in year 2. In addition, we expected that more study time in year 2 would lead to higher performance scores in year 2. Finally, we expected an interaction between time_Year 2_ and performance_Year 1_, with study time having increased effects on students with low-performance scores in year 1, compared to students with high-performance scores in year 1.

In a final analysis (analysis 3), we investigated whether a beneficial effect of study time could have long-lasting effects. In addition, we sought to investigate the effect of the quality of study time. More precisely, it may be that an overall positive effect of study time is due to some students who increased their study time and actually studied their learning material while another proportion of students increased their study time, but simply sat in front of their study material without actually studying. To investigate this, we measured the quality of study time as the study time that led to increased performance scores within the same year. A linear regression model with the independent variables performance_Year 1_ (interval scaled), the performance_Year 2_ (interval scaled), and time_Year2_ (interval scaled) and the dependent variable performance_Year 3_ (interval scaled), with all main effects and all interaction effects of these independent variables, was carried out to test the hypotheses. We expected that the amount of study time in year 2 (2018-2019) influenced the performance score throughout the following school year (2019-2020). In addition, we expected a significant three-way interaction showing students with low-performance scores in school year 1 (2017-2018), with increasing study time in year 2 (2018-2019) and increasing performance scores in year 2 (2018-2019), revealing increased performance scores in year 3 (2019-2020). However, low-performing students in school year 1 (2017-2018), with increased study time in year 2 (2018-2019) but no increase in performance in the second school year, were expected to remain on low-performance scores in the third school year (2019-2020).

## Results

**Fig. 1:**
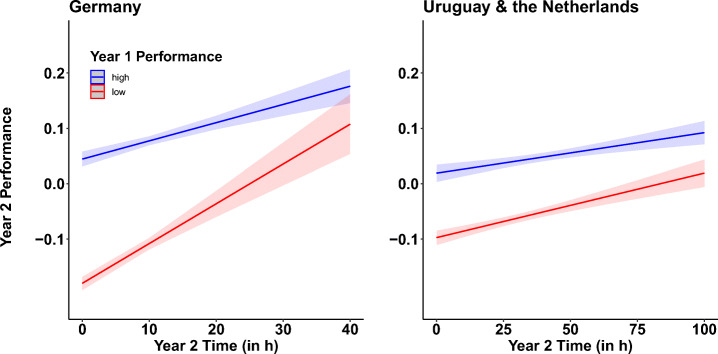
Year 2 performance in mathematics as a function of study time in hours in year 2 and performance in year 1. The more hours students studied in year 2, the higher the performance scores. This effect was more severe for students with weak performance scores in year 1 compared to students who already performed on a high level in year 1. The left panel graphs the results of German students. The right panel graphs the results of the combined datasets of Uruguayan students and students from the Netherlands. Blue-colored lines and shades indicate the model fits and standard error of the high quintile with the top 20% students of year 1. Red-colored lines and shades indicate the model fits and standard error of the low quintile with the weak 20% of the students of year 1

**Fig. 2:**
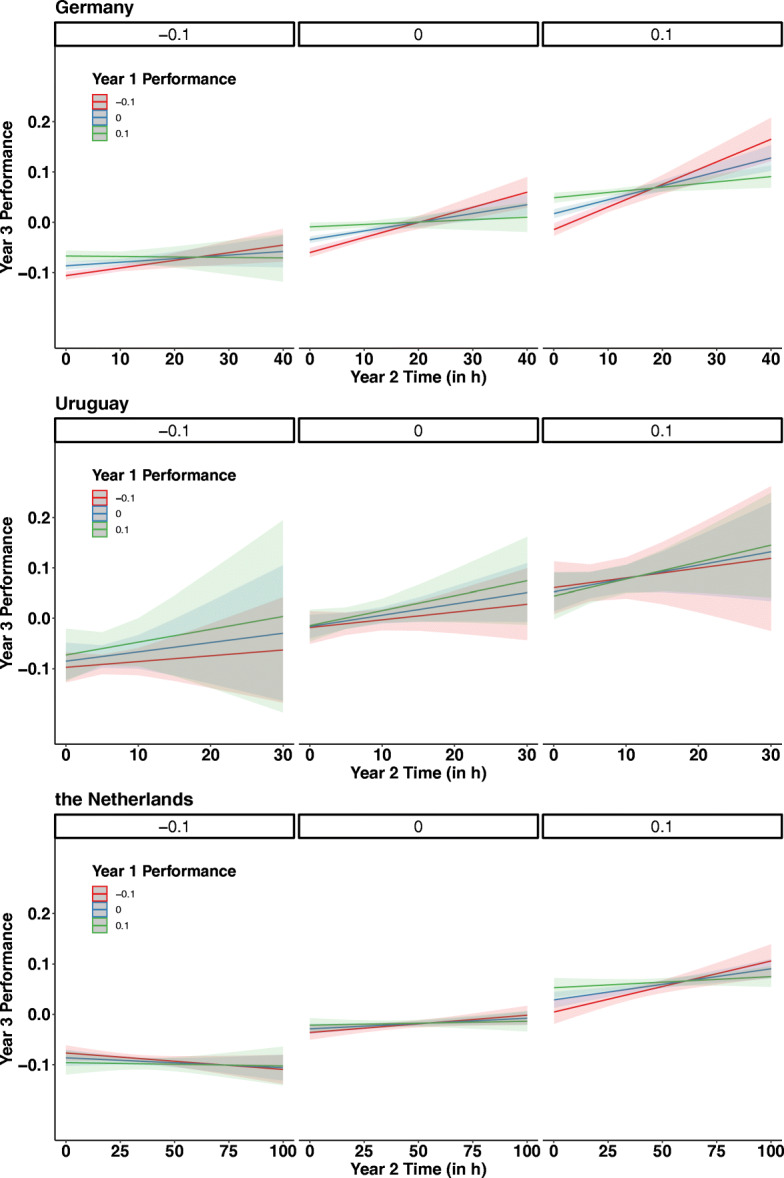
Year 3 performance in mathematics as a function of study time in hours in year 2, performance in year 1 (shown in colors for the three performance scores −.1, 0, .1) and performance in year 2 (shown in panels for the three performance scores −.1, 0, .1) for Germany (upper graph), Uruguay (middle graph), and the Netherlands (bottom graph). The more hours students spent to compute problem sets in year 2, the higher the performance in mathematics; however, if students’ performance scores increased in year 2, the study time spent was associated with steeper increases in performance scores (e.g., comparison between slope of red line on the left panel and red line on the right panel). Solid lines indicate the regression fits. Colored shades indicate the standard error of the mean

**Fig. 3:**
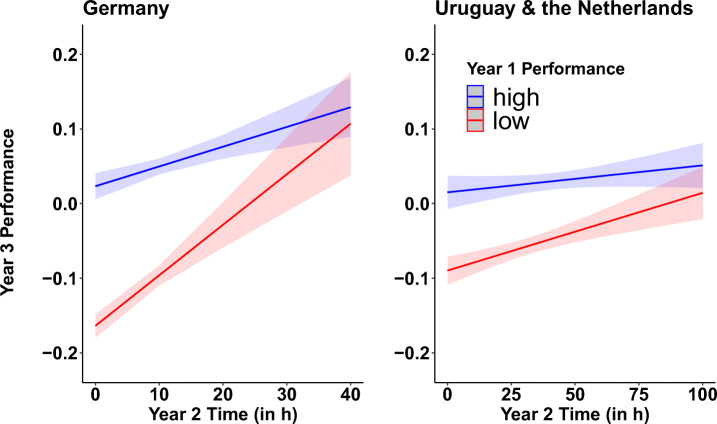
Students’ performance results of year 3 as a function of the extreme performance groups (quintiles) of year 1, and study time of year 2 for Germany (left) and the other two countries (right). Weak students who spent more time with the software revealed steeper performance gains in year 3 from more study time in year 2, compared to students who performed on high levels in year 1. Colored lines and shades indicate the model fits and standard errors, respectively

A summary of the descriptive statistics with the average study time and the average performance of each country and each school year, respectively, is provided in Table 2. The results of analysis 2 are depicted in Fig. 1 and results of analysis 3 are summarized in Fig. 2 and in Fig. 3.

**Table 2:** The average time in hours (time), standard deviation of time (time SD), average performance (performance), and standard deviation of performance (performance SD) for each country, and school year (year)

Country	Year	Time	Time SD	Performance	Performance SD
Germany	2017-2018	6.15	7.01	−0.01	0.12
Germany	2018-2019	8.59	7.55	−0.02	0.13
Germany	2019-2020	3.64	6.18	−0.03	0.16
Uruguay	2017-2018	5.56	6.20	−0.01	0.12
Uruguay	2018-2019	7.80	6.18	−0.01	0.09
Uruguay	2019-2020	1.44	1.83	−0.01	0.15
The Netherlands	2017-2018	22.07	27.65	< .01	0.09
The Netherlands	2018-2019	43.83	20.95	< .01	0.08
The Netherlands	2019-2020	26.20	20.72	−0.1	0.10

### Analysis 1: performance_Year 1_ as a function of time_Year 1_

The performance of students in the first school year (2017-2018) significantly increased with increasing study time in this school year (Germany: *b* = 0.004; *t*(4088) = 13.88; *p* < .001), Uruguay (*b* = .003; *t*(349) = 3.02; *p* = .002), and the Netherlands (*b* = .0002; *t*(1688) = 2.36; *p* = .018).

### Analysis 2: performance_Year 2_ as a function of performance_Year 1_ and time_Year 2_

Students’ performance during the first school year (2017-2018) significantly predicted their performance in the year after (Germany: *b* = .44; *t*(4086) = 17.11; *p* < .001; Uruguay: *b* = .41; *t*(347) = 4.25; *p* < .001; the Netherlands: *b* = .17; *t*(1686) = 3.34; *p* < .001). Moreover, the performance in year 2 significantly increased with increasing study time during the second year (Germany: *b* = .004; *t*(4068) = 11.67; *p* < .001; Uruguay: *b* = .004; *t*(347) = 3.68; *p* < .001; the Netherlands: *b* = .0008; *t*(1686) = 6.84; *p* < .001). The interaction between performance_Year 1_ and time_Year 2_ was not significant in any of the three analysis (Germany: *b* = −.003; *t*(4086) = −1.17; *p* = .238; Uruguay: *b* = −.002; *t*(347) = −0.18; *p* = .851; the Netherlands: *b* = .001; *t*(1686) = 1.18; *p* = .236). The lack of a significant interaction effect does not allow to conclude whether low-performing students in year 1 benefit more or less (in terms of performance in year 2) from extra study time in year 2 compared to high-performing students in year 1.

The non-significant interaction effect was further explored with two extreme performance groups of the first year: a low-performance group (bottom 20% of the performance students in year 1) and a high-performance group (upper 20% of the performance students in year 1). This quintile split was done for each of the three datasets. We used the German dataset for an exploratory analysis and aimed to replicate the observation made in this dataset with a combined dataset of Uruguayan and Dutch students^3^. Results indicated a significant interaction with a positive effect of more study time in year 2 on the performance in year 2, but a steeper increase of study time for those German students who performed low in year 1, compared to high-performance German students in year 1 (*b* = −.002; *t*(1632) = −4.12; *p* <.001). The combined dataset only revealed a trend towards this effect (*b* = −.0002; *t*(813) = −1.79; *p* = .073; see Fig. 1).

### Analysis 3: performance_Year 3_ as a function of performance_Year 1_, time_Year 2_, and performance_Year 2_

The main effect of performance_Year 1_ was significant in Germany but not the other two countries (Germany: *b* = 0.255; *t*(4082) = 8.23; *p* < .001; Uruguay: *b* = 0.019; *t*(343) = 0.17; *p* = .85; the Netherlands: *b* = 0.072; *t*(1682) = 1.40; *p* = .159), suggesting that the performance of students in the first year predicted their performance 2 years later in Germany. The lack of a significant result in the other two countries may be due to the smaller size of the dataset. The main effect of performance_Year 2_ on performance_Year 3_ was significant in all three countries (Germany: *b* = 0.517; *t*(4082) = 19.68; *p* < .001; Uruguay: *b* = 0.687; *t*(343) = 4.60; *p* < .001; the Netherlands: *b* = 0.575; *t*(1682) = 9.06; *p* < .001), suggesting that students’ performance in year 2 predicted their performance in year 3. The main effect of time_Year 2_ on performance_Year 3_ was significant in Germany, but we only observed a trend of this result in the other two countries (Germany: *b* = 0.001; *t*(4082) = 5.29; *p* < .001; Uruguay: *b* = 0.002; *t*(343) = 1.78; *p* = .075; the Netherlands: *b* = 0.0002; *t*(1682) = 1.87; *p* = .061). The interaction of performance_Year 1_ and time_Year 2_ was significant in Germany but not the other two countries (Germany: *b* = −0.012; *t*(4082) = −3.88; *p* < .001; Uruguay: *b* = 0.007; *t*(343) = 0.59; *p* = .550; the Netherlands: *b* = −0.001; *t*(1682) = −1.16; *p* = .243), with a positive effect of more study time for all students, but an increased effect of more study time in year 2 for low-performing students compared to high-performing students. This result was further explored with a quintile split as in analysis 2 (see below). The interaction of time_Year 2_ and performance_Year 2_ on the effect of performance_Year 3_ was significant in Germany and the Netherlands but not Uruguay (Germany: *b* = 0.60; *t*(4082) = 5.42; *p* < .001; Uruguay: *b* = −1.02; *t*(343) = −1.29; *p* = .197; the Netherlands: *b* = 1.68; *t*(1682) = 3.79; *p* < .001), with a positive effect of study time in year 2 on the performance in year 3, but an increased effect of study time in year 2 on year 3 for high-performance students in year 2 compared to low-performance students in year 2. The three-way interaction was significant in Germany (*b* = −.04; *t*(4082) = −2.70; *p* = .006), and the Netherlands (*b* = −.03; *t*(1682) = −2.28; *p* = .022), but not in Uruguay (*b* = .0005; *t*(343) = .005; *p* = .996; see Fig. 2). The three-way interaction indicated that low-performing students of year 1 who spent a lot of time studying mathematics in year 2 and showed severe improvements in year 2 continued to perform at a high level in year 3. However, low-performing year 1 students who spent more time using the software in year 2, but did not improve in year 2, did not show high-performance scores in year 3. In contrast to low-performance students, high-performance students showed smaller effects of more high-quality study time in year 2. This result showed that not only study time, but study time and improving while studying—i.e., the quality while studying, improved performance scores—was most beneficial for low-performance students in year 1.

The two-way interaction of performance_Year 1_ and time_Year_ 2 on performance_Year 3_ was further examined in an exploratory analysis (see Fig. 3). As in analysis 2, we conducted a quintile split with respect to the performance in year 1 for Germany and the combined dataset for the other two countries, respectively. Results showed a significant interaction in Germany (*b* = −.002; *t*(1632) = −3.38; *p* < .001) and the combined dataset (*b* = −.0003; *t*(813) = −1.97; *p* = .048) with more study time in year 2 leading to increased performance scores in year 3; however, low-performing students in year 1 revealed a steeper increase in performance in the third year, with more study time, compared to high-performing students.

## Discussion

The aim of this study was to systematically investigate the effect of study time spent on solving mathematical problem sets on students’ achievements in mathematics. Data collected with an online educational software for mathematics was used to address this question. More than six thousand K-12 students who computed more than 1.1 million problem sets within three consecutive school years were analyzed. Results showed that (a) study time had a positive effect on mathematical achievement scores of K-12 students (age 9-16); (b) especially low-performing students enhanced their performance with increased study time; (c) performance increases persisted over time; and (d) the quality of study time influences the effect of study time on performance scores. These results contribute to previous findings on whether study time influences achievement gains in mathematics (Cheema and Sheridan, 2015; Cooper et al., 2006; Cooper and Valentine, 2001; Gortner-Lahmers and Zulauf, 2000; Jez and Wassmer, 2015; Louw et al., 2008; Macnamara et al., 2014; Plant et al., 2005; Schuman et al., 1985; Trautwein, 2007) and replicated the observation of relatively stable academic abilities over time of previous studies (Aunio and Niemivirta, 2010; Jordan et al., 2011; Siegler et al., 2012; Watts et al., 2014). On the methodological consideration, we add to these studies by (a) using precise study time measures instead of assessing study time with questionnaires; (b) investigating the effects over a considerably large time frame of three consecutive school years; and (c) analyzing a large dataset.

Results from this study extend previous studies on the influence of study time on academic achievement scores by investigating whether the effects of study time depended on students’ prior abilities. A significant interaction of an exploratory analysis of analysis 2 provided evidence that extra study time in year 2 (2018-2019) led to high gains in performance for those students with the lowest 20% performance scores in year 1, compared to those students with the highest 20% performance scores in year 1^4^. This interaction was further backed up with an analysis (analysis 3) which investigated the interaction of study time in year 2 and performance in year 1 on students’ performance in year 3 and showed that the low-performing students (bottom 20%) of year 1 who increased their study time in year 2 revealed steep performance gains with increasing study time compared to high-performance students (upper 20%) of year 1. Together, these results provide evidence of the positive effect of extra study time on students’ performance and that especially weak students may boost their performance in mathematics the most by increasing their study time.

This investigation did not address the question of whether cognitive abilities (Hilbert et al., 2019), motivational factors (Perels et al., 2009; Rosário et al., 2013), or effort (Flunger et al., 2015; Trautwein, 2007) determine academic achievement scores. However, without spending time to learn something, by any means, how should learning occur at all? In other words, without increasing the quantity of study time, qualitative factors, such as cognitive abilities, motivational factors, or any psychological process with an effect on learning, always must have a limited effect on performance scores in mathematics. Nevertheless, we investigated the influence of times’ quality. The finding of analysis 3 suggests that students with low abilities in the first year who spent more time in the second year studying, and improved while studying, revealed high-performance scores in the third year, compared to those students who increased their study time, but did not improve while studying. This result shows that students who improve while studying (i.e., high-quality study time) reveal long-lasting effects of their study time. However, we propose that increasing their study time was an essential condition for learning in the first place and that other factors such as improving while studying, self-regulatory skills, motivational factors, and effort bear upon the study time spent learning. In short, the first condition which needs to be met in learning is the investment of time.

One possible methodological improvement in this study is the measurement of study time. While most of the previous studies assessed study time with questionnaires, this investigation measured study time using the computer software. Computer logs of study time provide more precise measures and are not biased through students’ biases regarding self-monitoring. As self-regulatory skills are known to influence academic achievement scores, such biases may directly influence results from studies using self-assessments. For example, it may be that students with elevated self-regulatory skills estimate their study time differently compared to students with low self-regulatory skills. Moreover, studies that examined study time with questionnaires did not examine the precise measure of study time with problem sets but study time overall. This may consist of recapping the previous lecture or lectures and may consist of much more time involvement than the actual study time spent to compute problem sets. Therefore, the accumulated study time or homework time measured with questionnaires may comprise much more study activities than the computation time of problem sets. Here, we investigated the effect of study time on the performance of the exercises the study time was measured and the performance on exercises later on, with positive effects of extra study time on both time scale measures.

The results of the present study are limited in several ways. First, Dutch students spent the most time with the software and the effect of extra study time might be lower for children who already studied a lot. Second, the time students spent with other learning materials, in addition to the software, was unknown, as well as teachers’ instructional time on topics in school. Both factors may influence the effect of study time on students’ performance. For instance, the effect of study time with the software may differ between students who spent less time, compared to more time, with paper and pencil problem sets. Third, students’ performance was measured as the deviation of their results to the results of other students on the same problem set. However, it may be that teachers assigned hard problem sets, which were above their abilities and the general learning goals, specifically to their strong students. In such a scenario, and if the actual strong student would not be able to reach a high accuracy on these problem sets, an actually strong student would reveal a low-performance score. Even though such a scenario cannot be ruled out in this current investigation, it appears that it does not reflect teachers’ usual assignment policy. More plausibly, teachers assign problem sets to their students which meet their standards with the aim of achieving a learning goal. Fourth, it is unclear which factors influenced the quality of study time. Unfortunately, the dataset did not include psychological factors such as motivation, or self-regulation, nor did it include differences between the characteristics of problem sets.

## Conclusion

These results may encourage poor performing students and their teachers to keep an eye upon engaging in mathematics and keep spending time to solve problem sets. High-school mathematics consists of comparably problems with clear solutions. In fact, all of these are solvable—in stark contrast to many math problems. The investment of time to seek out for the right answer is the first step to get it right.

## Footnotes

1 After the data was obtained and analyzed, the completion rate of finished problem sets of the students included in the analyses was provided for each cohort, respectively. The German cohort completed 97% of their problem sets, the Uruguayan cohort completed 94% of their problem sets, and the Dutch cohort completed 98% of their problem sets.

2 Please note that data of year 3 only considered problem sets computed between September 1st, until March 1st, due to the COVID-19 outbreak and the subsequent shutdown of schools.

3 The datasets were combined to keep the power high for this analysis.

4 Please note that this interaction was observed in the German dataset while the result of the other analysis that included the Uruguayan and Dutch students revealed a trend of this effect.
